# gSG6-P1 salivary biomarker discriminates micro-geographical heterogeneity of human exposure to *Anopheles* bites in low and seasonal malaria areas

**DOI:** 10.1186/1756-3305-6-68

**Published:** 2013-03-15

**Authors:** André Barembaye Sagna, Jean Biram Sarr, Lobna Gaayeb, Papa Makhtar Drame, Mamadou Ousmane Ndiath, Simon Senghor, Cheikh Saya Sow, Anne Poinsignon, Modou Seck, Emmanuel Hermann, Anne-Marie Schacht, Ngor Faye, Cheikh Sokhna, Franck Remoue, Gilles Riveau

**Affiliations:** 1 Centre de Recherche Biomédicale (CRB) Espoir Pour La Santé, 269 Route de la corniche, Sor - BP: 226, Saint-Louis, Sénégal; 2 Laboratoire de parasitologie générale, Département de Biologie Animale, Université Cheikh Anta Diop, Dakar, Sénégal; 3 Institut de Recherche pour le Développement, UMR 224 MIVEGEC, 911 avenue Agropolis - B: 64501, Montpellier, F-34394, France; 4 Centre d’Infection et d’Immunité de Lille (CIIL), Inserm U1019, CNRS UMR 8204, Université Lille Nord de France, Institut Pasteur de Lille, 1 rue du Pr. Calmette, Lille cedex, 59019, USA; 5 Institut de Recherche pour le Développement (IRD), UMR 198 URMITE Campus international de Hann, IRD – BP : 1386, Dakar, CP, 18524, Sénégal; 6 Institut de Recherche pour le Développement (IRD), UMR 224 MIVEGEC - Centre de Recherche Entomologique de Cotonou (CREC), Cotonou, Benin

**Keywords:** Malaria, Salivary peptide, Biomarker, Low transmission, *Anopheles* exposure, Antibodies

## Abstract

**Background:**

Over the past decade, a sharp decline of malaria burden has been observed in several countries. Consequently, the conventional entomological methods have become insufficiently sensitive and probably under-estimate micro-geographical heterogeneity of exposure and subsequent risk of malaria transmission. In this study, we investigated whether the human antibody (Ab) response to *Anopheles* salivary gSG6-P1 peptide, known as a biomarker of *Anopheles* exposure, could be a sensitive and reliable tool for discriminating human exposure to *Anopheles* bites in area of low and seasonal malaria transmission.

**Methods:**

A multi-disciplinary survey was performed in Northern Senegal where *An. gambiae s.l.* is the main malaria vector. Human IgG Ab response to gSG6-P1 salivary peptide was compared according to the season and villages in children from five villages in the middle Senegal River valley, known as a low malaria transmission area.

**Results:**

IgG levels to gSG6-P1 varied considerably according to the villages, discriminating the heterogeneity of *Anopheles* exposure between villages. Significant increase of IgG levels to gSG6-P1 was observed during the peak of exposure to *Anopheles* bites, and decreased immediately after the end of the exposure season. In addition, differences in the season-dependent specific IgG levels between villages were observed after the implementation of Long-Lasting Insecticidal Nets by The National Malaria Control Program in this area.

**Conclusion:**

The gSG6-P1 salivary peptide seems to be a reliable tool to discriminate the micro-geographical heterogeneity of human exposure to *Anopheles* bites in areas of very low and seasonal malaria transmission. A biomarker such as this could also be used to monitor and evaluate the possible heterogeneous effectiveness of operational vector control programs in low-exposure areas.

## Background

Improvement of diagnosis, treatment and preventive methods have brought about a sharp decrease of malaria transmission in several regions, particularly in Sub-Saharan Africa [1]. Over the past decade, several countries which formerly had a high malaria burden have seen over 50% reduction in malaria burden [2]. Consequently, the current methods for monitoring malaria have become increasingly difficult. Indeed, the evaluation of *Anopheles* population density is the first step to define the risk of transmission (Entomological Inoculation Rate, EIR) [3,4]. EIR estimates the number of infective bites a person receives per unit of time and thus the risk of exposure to malaria. However, the intensity of exposure to *Anopheles* bites, and thus the risk of malaria transmission, may be different from a local setting to another within a single micro-geographical region [5-7] and even between neighbouring villages or houses [8]. This heterogeneity of exposure to *Anopheles* is particularly important in areas of low malaria transmission, where only few infected mosquitoes are sampled and where focal hotspots of malaria transmission may exist [9]. These residual transmission foci may hamper elimination efforts by sending transmission to the wider community [10,11]. Moreover, the evaluation of the real exposure *to Anopheles*, and thus the risk of malaria transmission by the EIR, seems irrelevant and not adapted in these contexts because the number of collected mosquitoes are often below the detection limits of commonly used trapping methods [7,9]. It has been shown there is significant malaria transmission in the Senegal River Basin, yet entomological data showed a very low exposure to *Anopheles* bites [12,13]. People living in such settings could be at a high risk of malaria morbidity and mortality because of the absence of protective immunity due to low levels of parasite exposure. The development of simple, rapid and sensitive tools is therefore needed to identify the micro-geographical variations of exposure and thus the risk of transmission in areas of low or very low exposure to *Anopheles*. Those tools could be useful for targeting areas where the control should be strengthened.

Several studies have shown a relationship between human antibody (Ab) responses to whole arthropod saliva and the human exposure to triatomines [14,15], tsetse flies [16,17], sandflies [18-20], *Aedes* and *Culex*[21,22], and *Anopheles* species [23-25]. However, many areas exhibit several species of blood-sucking arthropods [26,27], therefore high specificity and sensitivity were needed to evaluate a specific arthropod exposure by salivary-based immunoassays. Indeed, many cross-reactions have been reported for whole saliva between different vectors and also between closely related species [28]. During the past 10 years, advances in the study of transcriptome and proteome of *Anopheles gambiae* (*An. gambiae*) identified gSG6, a small salivary protein specific to *Anopheles* species [29] and presenting antigenic properties. The whole gSG6 protein was detected by IgG Ab from children exposed to *An. gambiae* bites and was then proposed as a biomarker of exposure [30,31]. In order to optimize the gSG6 biomarker, Poinsignon *et al.*, by coupling bioinformatic and immuno-epidemiological approaches, identified one peptide of gSG6, the gSG6-P1 peptide, as a relevant and specific biomarker of *Anopheles* exposure [30]. The IgG response to this specific peptide is perfectly correlated to both human exposure to bites of *An. gambiae* and *An. funestus*[32]. In addition, it has been suggested that this biomarker was particularly suited to assess low-level exposure to *An. gambiae* bites [33]. Nevertheless, this biomarker has not been validated for discriminating micro-geographical variation of exposure in a low and seasonal malaria transmission area.

The present study aims to assess if the gSG6-P1 salivary peptide could be a sensitive tool for discriminating human exposure to *An. gambiae* bites in a micro-geographical context of low and seasonal malaria transmission. To this end, the specific IgG response to gSG6-P1 was evaluated during 1.5 years follow-up (rainy and dry seasons) in children living in five different villages in the middle Senegal River valley.

## Methods

### Study area and population

**Figure 1:**
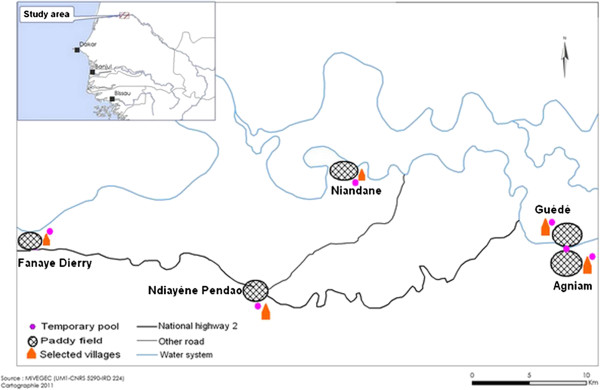
Localization of studied villages.

This study was carried out in Northern Senegal (Podor District) along the Senegal River Basin (Figure 1). The studied majority of the population belongs to the Peulh ethnic group. This region is a dry savannah, with a dry season from November to June and a short rainy season from July to October (annual rainfall <400 mm in 2009) [34]. In this region, malaria transmission is very low, seasonal and mainly due to *An. gambiae s.l.*[35].

A longitudinal survey was performed in five villages (Agniam, Niandane, Ndiayene Pendao, Guede and Fanaye) and five visits (October 2008, January, June, October 2009 and January 2010) were carried out. The study cohort consisted of 410 children aged from 1 to 9 years, but only the children present and blood sampled at each of the 5 visits were included for the immunological analysis (n=265). Age mean at the beginning of the study differed between these five villages: Agniam (mean±SD): 4.40 ± 2.48, Niandane: 5.17 ± 2.42, Pendao: 4.49 ± 2.61, Guede: 5.84 ± 3.06 and Fanaye: 5.32 ± 2.46) (p=0.008). Thick blood smears were stained with Giemsa to identify *Plasmodium* species and the number of malaria parasites was counted. Parasite density was defined as the number of *Plasmodium* parasites/μl of blood. In parallel, sera collected by finger prick were used for immunological tests. In June 2009, a large scale distribution of Long-Lasting Insecticidal Nets (LLINs) was performed around the endemic regions, and particularly in the studied region by the National Malaria Control Program (NMCP) of Senegal [36].

The present study was approved by the National Ethics Committee of the Ministry of Health of Senegal, (October 2008; 0084/MSP/DS/CNRS, ClinicalTrials.gov ID: NCT01545115). Oral and written informed consents were obtained from the parents or the legal guardians of the children.

### Mosquito sampling and entomological analysis

Mosquito collection procedures and their treatment in the laboratory were previously described by Ndiath *et al.*[35]. Briefly, Human Landing Catches (HLC) were performed from 07:00 p.m to 07:00 a.m for two non consecutive nights. Four adult volunteer collectors were positioned at two different sites in each village (2 collected mosquitoes indoor and 2 outdoor). Pyrethrum Spray Catches (PSC) were conducted in five randomly selected rooms for one day among those not having used any form of insecticide or repellent during the previous week and being different from those used for HLC. Deltamethrin (Yotox^®^) was sprayed inside the closed rooms for 30–45 seconds. After 10 minutes, dead or immobilized mosquitoes were collected. *Anopheles* species were identified using morphological characteristics according to identification rules [37]. Human Biting Rate (HBR) was estimated by the number of *An. gambiae* bites/human/night (BHN) sampled by HLC. It was calculated by dividing the number of *An. gambiae* caught by the total person-night for the period. The density of *An. gambiae* females resting in a room was estimated by the number of *An. gambiae* Females per Room per Night (FRN) sampled by PSC. It was calculated by dividing the number of *An. gambiae* species identified by the total randomized-rooms for the period, as previously described [35].

### Salivary peptide gSG6-P1

The gSG6-P1 peptide was designed as previously described [30]. It was synthesized and purified (>95%) by Genepep SA (St-Clément de Rivière, France). Peptide was shipped in lyophilized form and then suspended in 0.22 μm ultra-filtered water and frozen at -80°C until use.

### Evaluation of human IgG antibody levels (ELISA)

ELISAs were carried out on sera to quantify IgGs to the gSG6-P1 peptide as previously described [38]. Briefly, the gSG6-P1 antigen (20 μg/mL) was coated onto Maxisorp plates (Nunc, Roskilde, Danemark) using 100 μL/well for 2 h 30 min at 37°C. Plate wells were then blocked for 1 h at room temperature with 300 μL of protein-free blocking buffer, pH 7.4 (Thermoscientific, Rockford, USA). Individual sera were incubated in duplicate at 4°C overnight at a 1/20 dilution (in PBS with 1% Tween). This dilution was determined as optimal after several preliminary experiments. Plates were then incubated for 90 min at 37°C with 100 μL of mouse biotinylated Ab against human IgG (BD Pharmingen, San Diego CA, USA) diluted 1/2000 in PBS with 1% Tween. Plate wells were then washed and incubated for 1 h at 37°C with 100 μL of peroxidase-conjugated streptavidin (Amersham, les Ulis, France). Colorimetric development was carried out using ABTS (2.2'-azino-bis (3 ethylbenzthiazoline 6-sulfonic acid) diammonium; Sigma, St Louis, MO, USA) in 50 mM citrate buffer (Sigma, pH = 4, containing 0.003% H2O2) and absorbance (OD) was measured at 405 nm.

Individual results were expressed as the ΔOD value: ΔOD = ODx­ODn, where ODx represents the mean of individual optical density (OD) value in both wells with gSG6-P1 antigen and ODn the individual OD value for each serum without gSG6-P1 antigen. Anti-gSG6-P1 IgG levels were also assayed in non-*Anopheles* exposed individuals (n = 12 – neg; North of France) in order to quantify the non-specific background Ab level and to calculate the specific immune response threshold (TR): TR = mean (ΔOD_neg_) + 3SD = 0.180. An exposed individual was then classified as an immune responder (IR) if its ΔOD> 0.180.

### Statistical analysis of data

Data were analysed with Graph Pad Prism^®^ (Graph Pad Software, San Diego, USA). After checking that values in each group did not fit a Gaussian distribution, one-way analysis of variance (ANOVA) was used to compare age differences between children of all villages. The non-parametric Mann–Whitney U test was used to compare Ab response levels between two villages while the Kruskal-Wallis test was used for the comparison of Ab response levels between more than two villages. The Wilcoxon matcher-paired test was used for the comparison of Ab response levels between two visits in each village. All differences were considered as significant at p<0.05.

## Results

### Entomological and parasitological data

Previous results indicated that malaria transmission in the study villages was low and seasonal with an EIR (number of infective bites/person/night) ranging from 0 to 0.059 [35]. The prevalence of *P. falciparum* infection was also low to moderate (ranging from 0 to 31.7%), season-dependent and peaked in January 2009, after the peak of *Anopheles* exposure (Table 1). However, malaria prevalence in October 2009 was very low compared to October 2008 in all studied villages. Such a decrease may be related to the implementation of LLINs during the end of the dry season (June 2009) by NMCP in the studied area. In studied villages, the large majority (80%) of anopheline species belong to the *An. gambiae* complex, as previously reported [35]. *Anopheles* density (BHN and FRN) was generally low and variable according to the village (Table 1). Whatever the considered entomological parameters (BHN or FRN), the higher density was generally observed in Agniam, Niandane and Pendao villages compared to Guede and Fanaye villages. The FRM results indicated however, that Agniam could be considered as the village presenting the highest exposure risk to *An. gambiae* exposure, compared to other villages. A marked increase in *Anopheles* density was observed during both rainy seasons (October 2008 and October 2009) compared to the dry season (January through June 2009) in all studied villages except Pendao in October 2009.

**Table 1:** Characteristics of the studied population: entomological, parasitological and immunological data

**Villages**	**Variables**	**Periods of survey**				
		**October 2008**	**January 2009**	**June 2009**	**October 2009**	**January 2010**
Agniam (n=41)	*An. gambiae *Bites/human/night	8.37	0	1.62	12.5	0.5
	*An. gambiae *Females/Room/night	41	0.2	7.6	28.4	4.4
	% *P. falciparum *prevalence (95% CI)a	12.20 (2.18 ; 22.22)	19.50 (7.38 ; 31.64)	4.90 (-1.77 ; 11.47)	0.00	2.40 (-2.28 ; 7.16)
	% of immune responders (95% CI)b	80.49 (68.36 ; 92.62)	34.15 (19.63 ; 48.67)	29.27 (15.34 ; 43.2)	82.92 (71.41 ; 94.45)	24.40 (11.25 ; 37.53)
Niandane (n=71)	*An. gambiae *Bites/human/night	13.87	0.62	1.5	11.12	0
	*An. gambiae *Females/Room/night	11.6	0.6	1.4	9.4	0
	*% P. falciparum *prevalence (95% CI)	21.10 (11.63 ; 30.63)	24.00 (14.01 ; 33.87)	4.20 (-0.45 ; 8.91)	4.20 (-0.45 ; 8.91)	0.00
	% of immune responders (95% CI)	54.93 (43.36 ; 66.5)	21.12 (11.63 ; 30.63)	18.30 (9.30 ; 27.30)	33.80 (22.8 ; 44.8)	14.08 (5.99 ; 22.17)
Pendao (n=37)	*An. gambiae *Bites/human/night	9.25	0.87	3.5	0.87	0.62
	*An. gambiae *Females/Room/night	20.4	0.6	7.6	4.8	0.2
	*% P. falciparum *prevalence (95% CI)	5.40 (-1.88 ; 12.7)	16.20 (4.34 ; 28.1)	2.70 (-2.52 ; 7.92)	0.00	0.00
	% of immune responders (95% CI)	37.84 (22.21 ; 53.47)	24.32 (10.5 ; 38.14)	10.81 (0.8 ; 20.82)	32.43 (17.37 ; 47.51)	8.11 (-0.69 ; 16.91)
Guede (n=41)	*An. gambiae *Bites/human/night	7.12	0	0.25	9.37	1.75
	*An. gambiae *Females/Room/night	9.8	0	3	7.8	3.6
	*% P. falciparum *prevalence (95% CI)	9.75 (0.68 ; 18.84)	31.70 (17.47 ; 45.95)	7.30 (-0.65 ; 15.29)	0.00	4.90 (-1.71 ; 11.47)
	% of immune responders (95% CI)	39.02 (24.09 ; 53.95)	7.32 (-0.65 ; 15.29)	21.95 (9.28 ; 34.62)	43.90 (28.71 ; 59.09)	12.19 (2.18 ; 22.22)
Fanaye (n=75)	*An. gambiae *Bites/human/night	2.75	0	0.75	3.87	0.37
	*An. gambiae *Females/Room/night	7.8	1.4	1.2	7.4	1.2
	*% P. falciparum *prevalence (95% CI)	16.00 (8.76 ; 25.9)	18.70 (9.85 ; 27.49)	2.70 (-0.98 ; 6.32)	1.30 (-1.26 ; 3.92)	0.00
	% of immune responders (95% CI)	9.33 (2.75 ; 15.91)	0.00	1.33 (-1.26 ; 3.92)	0.00	1.33 (-1.26 ; 3.92)

n= number of children;

Bites/human/night= number of *An. gambiae *caught by Human Landing Catches;

Females/Room/Night= number of *An. gambiae* females caught by Pyrethrum Spray Catches;

a Lower and upper 95% confidence interval of *Plasmodium falciparum *prevalence.

b Lower and upper 95% confidence interval of Immune responders to gSG6-P1 salivary peptide.

### IgG response levels to gSG6-P1 according to age group

Specific IgG responses to *An. gambiae* s.l. gSG6-P1 peptide were analyzed in October 2008 (period of higher exposure to Anopheles) in children aged 1 to 9 years according to age groups (1–2, 3–4, 5–6, 7–8 and 9 years old). Cumulative immunological data from all villages indicated that only 44% of children were immune responders. The median of IgG response to gSG6-P1 differed significantly according to age groups (p=0.039). Specific IgG level was low in children from the 1–2 years age group, increased in the 3–4 years age group, and then remained high from this age to 9 years old (data not shown).

### IgG response levels to gSG6-P1 according to the village

Anti-gSG6-P1 IgG levels were compared in the five studied villages at the peak period of exposure to *Anopheles* bites (October 2008, Figure 2). Despite the inter-individual heterogeneity observed in each village, the median of specific IgG Ab levels in children varied significantly according to villages (p<0.0001). Children from Agniam (A) developed significantly higher IgG response levels to gSG6-P1 than those from other villages (p<0.0001 compared to each village value). In contrast, no significant differences in specific IgG levels were found between children from villages of Niandane (N), Pendao (P) and Guede (G). IgG Ab levels to gSG6-P1 were significantly lower in children from Fanaye compared to the IgG levels of those from other villages (every p<0.0001). These differences of anti-gSG6-P1 IgG levels according to the studied villages were also observed whatever the considered age-group (1–5 or 6–9 years old age groups, data not shown). In the same way, the percentage of immune responders was high in Agniam (80.49%), moderate in Niandane (54.93%), Pendao (37.84%) and Guede (39.02%) and very low in Fanaye (9.33%) (Table 1). Taken together, these results indicated that the specific IgG levels and the percentage of immune responders to gSG6-P1 peptide were village-dependent.

**Figure 2:**
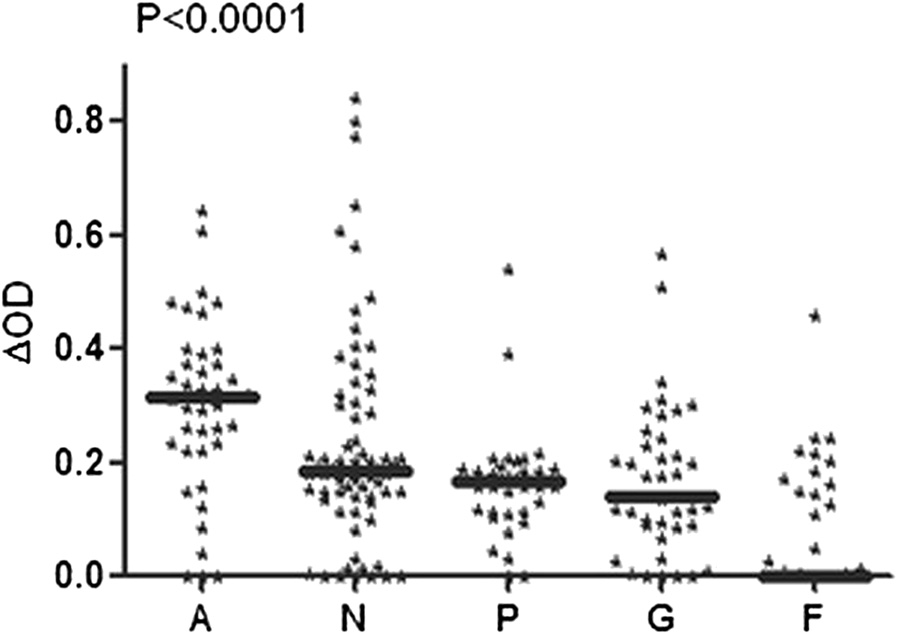
**IgG Ab levels to gSG6-P1 according to the village. **Individual IgG response levels are presented at the peak of malaria transmission (October 2008) in studied villages: Agniam (A), Niandane (N), Pendao (P), Guede (G) and Fanaye (F). Bold lines represent ΔOD median values. P value of the Kruskal-Wallis U test is indicated.

However, some inconsistencies were observed between immunological and entomological results in some villages and seasons accordingly. Indeed, a low percentage of immune responders could be observed in villages and/or seasons where high HBR were detected and vice versa (Table 1). For instance, in Fanaye, the percentage of immune responders was nil while entomological data showed a rate of 3.87 BHN, in October 2009. Likewise, in October 2009 in Guede, BHN was 9.37 and the percentage of immune responders was 43.9%, while similar levels of biting rates of 8.37 BHN in October 2008 in Agniam showed a higher rate of immune response at 80%.

### Seasonal variation of the IgG levels to gSG6-P1 peptide

**Figure 3:**
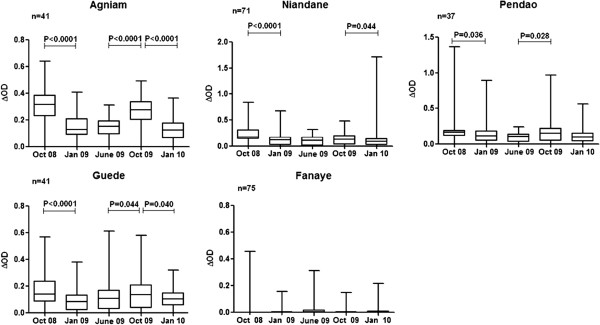
**Seasonal variation of the IgG levels to gSG6-P1 peptide. **IgG response levels of children present at all visits are considered. Individual IgG responses (ΔOD) to gSG6-P1 peptide are presented and bars indicate the median value for studied individuals in each season, and according to the studied villages (Agniam, Niandane, Pendao, Guede and Fanaye). The boxes locate the middle 50% of the data; horizontal lines in the boxes indicate medians of the data; lengths of boxes correspond to the inter-quartile ranges. P value of the Wilcoxon matched-paired test is indicated only if significant (p<0.05).

The IgG response levels to gSG6-P1 peptide were evaluated in each village during a 16 months follow-up (Figure 3). As a general pattern, the anti-gSG6-P1 IgG levels were significantly higher during the period of high exposure to *Anopheles* (October 2008; rainy season) compared to other periods. The IgG levels then decreased significantly at the beginning of the dry season (January 2009) (p<0.05 in all villages, except Fanaye) and remained low until the end of this season. In October 2009, this season-dependent variation of the IgG levels to gSG6-P1 was only observed in Agniam, Pendao and Guede but not in Niandane where specific IgG responses remained low compared to June 2009 (dry season) (p=0.147). In Fanaye, a very low IgG response level was observed regardless of the period studied. A similar seasonal-dependent variation was observed for the percentage of immune responders (Table 1) where the number of responders was higher during October 2008 than in June 2009.

Furthermore, specific IgG levels to gSG6-P1 in October 2009 were lower compared to those recorded in October 2008, only in children living in Agniam and Niandane villages (p<0.0016 and p<0.0001, respectively). Entomological data indicated similar densities of Anopheles between October 2008 and October 2009 in Agniam and in Niandane villages (Table 1). No significant differences in IgG levels between October 2008 and 2009 were observed in the three other villages.

## Discussion

In the context of low malaria transmission, the current methods used to evaluate the intensity of transmission, such as EIR or *Plasmodium* parasitemia, present substantial limitations. Alternative methods to estimate *Anopheles* density and human exposure would be of great value, allowing epidemiological studies when the use of classical methods may not be relevant such as in low transmission settings. In this respect, our present study investigated whether the gSG6-P1 salivary peptide could be a sensitive and reliable biomarker allowing the detection of micro-geographical heterogeneity of human exposure to *Anopheles* bites in particular settings.

Our results showed that the IgG Ab levels to gSG6-P1 peptide and the percentage of immune responders varied between the five studied villages. These results suggest that the immune response to gSG6-P1 salivary peptide could identify villages more at risk of malaria than others even in an area presenting low exposure to vectors. The high heterogeneity of exposure to *Anopheles* bites observed in the studied villages may be explained by the presence of different surrounding landscapes among them [39,40] and/or the proximity of the river. Indeed, several studies have shown a positive correlation between malaria transmission and the distance to a river as a potential breeding site [41,42]. Nevertheless, this factor is not sufficient to explain the variation of *Anopheles* exposure between villages. In fact, Agniam, Guede and Niandane, three villages located near the river, presented different levels of IgG responses to gSG6-P1. A possible explanation of the high human-*Anopheles* contact in Agniam compared to Guede, for instance (villages only separated by the river) could be the influence of man-made conditions. In this area, Ndiath *et al.* have shown that *Anopheles* density variation was more related to the presence of ditch water used for gardening, rice cultivation, manufacture of bricks or animal watering, than the proximity of the river [35]. Thus, *Anopheles* exposure could be influenced by local human activity and/or household factors which may favor the development of artificial breeding sites, allowing the emergence of adults [35]. Overall, these results confirm that the gSG6-P1 peptide could measure the real human-vector contact and is sensitive enough to detect small-scale variations of vector bites in areas with very low-exposure [32,33,43].

However, inconsistencies observed between immunological parameters and entomological results in some villages (e.g.: Agniam vs. Guede) and/or according to seasons suggest that other factors could be taken into account in measuring the heterogeneity of man-vector contact. The use of vector control tools (spray, ITNs or LLINs, personal protection…) as well as household characteristics (traditional, modern…) could significantly reduce the human-vector contact. Moreover, individual exposure to *Anopheles* was evaluated by HLC (using adult volunteers) and therefore, could present considerable limitations for evaluation of entomological exposure in children [44,45]. Entomological data was also collected in limited number of areas (five randomised houses/village) and cannot represent the individual exposure to Anopheles bites and its micro-geographical variations. The use of the gSG6P1 biomarker could therefore be relevant and useful for assessing human exposure to *Anopheles* bites at the population and individual level.

We also reported that both IgG Ab levels and the percentage of immune responders to the salivary biomarker varied according to the season and remained high in October corresponding to the peak of exposure to *Anopheles* bites. This seasonal variation of specific IgG response was associated to the similar variations of entomological data, as has been reported in previous studies [31,38,46]. Moreover, the drop of IgG response observed in January 2009, only three months after the peak of exposure (October 2008), confirmed that the anti-gSG6-P1 IgG responses were short-lived, decreasing after a few months of no exposure [33,47]. These observations indicate that the Ab responses to gSG6-P1 antigen are transient and sensitive to the seasonal variations of human exposure to *Anopheles* bites. Similar seasonal fluctuations of Ab to gSG6-P1 were reported in some other epidemiological studies taking place elsewhere in Africa [33,47]. Indeed, a drop in the anti-saliva IgG response has been previously described in soldiers exposed to *An. gambiae*, three months after their return from a travel period in tropical Africa [48] and in children from Angola 6 weeks after the interruption of exposure by bednet use [47]. This concomitant variation of IgG response according to season and consequently to *Anopheles* exposure indicates that the gSG6-P1 biomarker could be used as an alternative tool when trapping methods are difficult to apply, are fastidious and not applicable at large scale [49,50], particularly in low or very low endemic areas.

In addition, we observed village-dependent differences between 2009 and 2008. Indeed, specific IgG Ab levels to gSG6-P1 significantly increased only in Agniam, Pendao and Guede in October 2009 compared to June 2009, but not in Niandane village. Moreover, specific IgG responses were lower in October 2009 compared to October 2008 in Agniam and Niandane, whereas entomological data indicated that *Anopheles* populations remained similar at these two time periods. As gSG6-P1 biomarker has clearly been shown as a pertinent indicator for measuring the efficacy of LLINs [36], the present data could indicate a change in human-vector contact between both periods. Indeed, human-vector contact may be influenced by several human or environmental factors [51]. The large scale implementation of LLINs conducted by the NMCP in June 2009 [36] could explain the reduced probability of being bitten by *Anopheles* and partly explain the observed immunological results. However, it is necessary to indicate that despite this implementation of LLINs, some individuals presented gSG6-P1 specific IgG responses indicating that they were still exposed to *Anopheles* bites. Altogether, we can hypothesize that the observed differences between villages could be due to a different distribution, owners or real use of LLINs by children according to villages. For instance, it could be hypothesized that the efficacy of LLINs implementation and use by children could be higher in Niandane village, and also in Agniam to a lesser extent, compared to other villages. Some factors such as genetics, nutritional status, population displacement (holidays spent in an area of low/high exposure for school-age children), micro-climatic and micro-habitat variations from the studied villages, could not be excluded to explain the observed differences. Nevertheless, these data suggest that the gSG6-P1 biomarker could represent an alternative tool for evaluating the effectiveness of vector control strategies by The National Malaria Control Programmes and variations of effectiveness between villages and environmental and epidemiological contexts [38,43]. Future studies evaluating the impact of LLINs on malaria transmission in children could be performed by a multi-disciplinary approach where immune response to salivary biomarker would be integrated.

## Conclusions

The measurement of human Ab responses to gSG6-P1 represents a new tool for evaluating human exposure to Anopheles vector bites. This specific Ab response seems to be sensitive, reliable and complementary to classical entomological methods used for evaluating the heterogeneity of human exposure to Anopheles bites, in areas with low-levels of malaria transmission. This biomarker could be used as a pertinent tool to estimate short term variations of vector exposure and as a promising indicator to evaluate the effectiveness of vector control strategies particularly in areas with low endemicity. In addition, a biomarker such as this would allow targeting for anti-malaria control programmes in specific areas and in seasons where malaria risk is highest.

## Competing interests

The authors declare that they have no competing interests.

## Authors’ contributions

ABS carried out the immunological assessments, statistical analysis and drafted the manuscript. LG and JBS helped to draft the manuscript. JBS, LG, MON, CB, SS, CSS, MS, contributed to field activities and microscopic examination. PMD, AP, EH, AMS, NF, CS provided substantial improvement of the manuscript. FR, GR provided the scientific supervision, interpretation of the data, and revised the manuscript. All authors approved the final version of the manuscript.

## References

[B1] WHO World Malaria Report 2011 2011 Geneva: World Health Organisation

[B2] O’Meara WP Mangeni JN Steketee R Greenwood B Changes in the burden of malaria in sub-Saharan Africa Lancet Infect Dis 2010 10 545 555 10.1016/S1473-3099(10)70096-7 20637696

[B3] Drakeley C Schellenberg D Kihonda J Sousa CA Arez AP Lopes D Lines J Mshinda H Lengeler C Armstrong Schellenberg J Tanner M Alonso P An estimation of the entomological inoculation rate for Ifakara: a semi-urban area in a region of intense malaria transmission in Tanzania Trop Med Int Health 2003 8 767 774 10.1046/j.1365-3156.2003.01100.x 12950662

[B4] Hay SI Rogers DJ Toomer JF Snow RW Annual *Plasmodium falciparum* entomological inoculation rates (EIR) across Africa: literature survey, Internet access and review Trans R Soc Trop Med Hyg 2000 94 113 127 10.1016/S0035-9203(00)90246-3 10897348 PMC3204456

[B5] Amek N Bayoh N Hamel M Lindblade KA Gimnig J Odhiambo F Laserson KF Slutsker L Smith T Vounatsou P Spatial and temporal dynamics of malaria transmission in rural Western Kenya Parasit Vectors 2012 5 86 10.1186/1756-3305-5-86 22541138 PMC3464956

[B6] Mbogo CM Mwangangi JM Nzovu J Gu W Yan G Gunter JT Swalm C Keating J Regens JL Shililu JI Githure JI Beier JC Spatial and temporal heterogeneity of *Anopheles* mosquitoes and *Plasmodium falciparum* transmission along the Kenyan coast AmJTrop Med Hyg 2003 68 734 742 12887036

[B7] Smith T Charlwood JD Takken W Tanner M Spiegelhalter DJ Mapping the densities of malaria vectors within a single village Acta Trop 1995 59 1 18 10.1016/0001-706X(94)00082-C 7785522

[B8] Ye Y Kyobutungi C Louis VR Sauerborn R Micro-epidemiology of *Plasmodium falciparum* malaria: Is there any difference in transmission risk between neighbouring villages? Malaria J 2007 6 46 10.1186/1475-2875-6-46 PMC1858701 17445255

[B9] Oesterholt MJ Bousema JT Mwerinde OK Harris C Lushino P Masokoto A Mwerinde H Mosha FW Drakeley CJ Spatial and temporal variation in malaria transmission in a low endemicity area in northern Tanzania Malaria J 2006 5 98 10.1186/1475-2875-5-98 PMC1635725 17081311

[B10] Bousema T Griffin JT Sauerwein RW Smith DL Churcher TS Takken W Ghani A Drakeley C Gosling R Hitting hotspots: spatial targeting of malaria for control and elimination PLoS Med 2012 9 e1001165 10.1371/journal.pmed.1001165 22303287 PMC3269430

[B11] Moonen B Cohen JM Snow RW Slutsker L Drakeley C Smith DL Abeyasinghe RR Rodriguez MH Maharaj R Tanner M Targett G Operational strategies to achieve and maintain malaria elimination Lancet 2010 376 1592 1603 10.1016/S0140-6736(10)61269-X 21035841 PMC3037542

[B12] Dia I Konate L Samb B Sarr JB Diop A Rogerie F Faye M Riveau G Remoue F Diallo M Fontenille D Bionomics of malaria vectors and relationship with malaria transmission and epidemiology in three physiographic zones in the Senegal River Basin Acta Trop 2008 105 145 153 10.1016/j.actatropica.2007.10.010 18068685

[B13] Sarr JB Remoue F Samb B Dia I Guindo S Sow C Maiga S Tine S Thiam C Schacht AM Simondon F Konate L Riveau G Evaluation of antibody response to Plasmodium falciparum in children according to exposure of Anopheles gambiae s.l or Anopheles funestus vectors Malaria J 2007 6 117 10.1186/1475-2875-6-117 PMC2008208 17764568

[B14] Nascimento RJ Santana JM Lozzi SP Araujo CN Teixeira AR Human IgG1 and IgG4: the main antibodies against *Triatoma infestans* (Hemiptera: Reduviidae) salivary gland proteins AmJTrop Med Hyg 2001 65 219 226 10.4269/ajtmh.2001.65.219 11561708

[B15] Schwarz A Sternberg JM Johnston V Medrano-Mercado N Anderson JM Hume JC Valenzuela JG Schaub GA Billingsley PF Antibody responses of domestic animals to salivary antigens of *Triatoma infestans* as biomarkers for low-level infestation of triatomines Int J Parasitol 2009 39 1021 1029 10.1016/j.ijpara.2009.01.010 19248784 PMC2748746

[B16] Caljon G Van Den Abbeele J Sternberg JM Coosemans M De Baetselier P Magez S Tsetse fly saliva biases the immune response to Th2 and induces anti-vector antibodies that are a useful tool for exposure assessment Int J Parasitol 2006 36 1025 1035 10.1016/j.ijpara.2006.05.002 16777113

[B17] Poinsignon A Remoue F Rossignol M Cornelie S Courtin D Grebaut P Garcia A Simondon F Human IgG antibody response to *Glossina* saliva: an epidemiologic marker of exposure to *Glossina* bites AmJTrop Med Hyg 2008 78 750 753 18458309

[B18] Barral A Honda E Caldas A Costa J Vinhas V Rowton ED Valenzuela JG Charlab R Barral-Netto M Ribeiro JM Human immune response to sand fly salivary gland antigens: a useful epidemiological marker? AmJTrop Med Hyg 2000 62 740 745 10.4269/ajtmh.2000.62.740 11304066

[B19] Marzouki S Ben Ahmed M Boussoffara T Abdeladhim M Ben Aleya-Bouafif N Namane A Hamida NB Ben Salah A Louzir H Characterization of the antibody response to the saliva of *Phlebotomus papatasi* in people living in endemic areas of cutaneous leishmaniasis AmJTrop Med Hyg 2011 84 653 661 10.4269/ajtmh.2011.10-0598 PMC3083729 21540371

[B20] Rohousova I Ozensoy S Ozbel Y Volf P Detection of species-specific antibody response of humans and mice bitten by sand flies Parasitology 2005 130 493 499 10.1017/S003118200400681X 15991492

[B21] Doucoure S Mouchet F Cornelie S Dehecq JS Rutee AH Roca Y Walter A Herve JP Misse D Favier F Gasque P Remoue F Evaluation of the human IgG antibody response to *aedes albopictus* saliva as a New specific biomarker of exposure to vector bites PLoS Negl Trop Dis 2012 6 e1487 10.1371/journal.pntd.0001487 22363823 PMC3283547

[B22] Peng Z Li H Simons FE Immunoblot analysis of IgE and IgG binding antigens in extracts of mosquitos *Aedes vexans*, *Culex tarsalis* and *Culiseta inornata* Int Arch Allergy Immunol 1996 110 46 51 10.1159/000237309 8645977

[B23] Estevez PT Satoguina J Nwakanma DC West S Conway DJ Drakeley CJ Human saliva as a source of anti-malarial antibodies to examine population exposure to *Plasmodium falciparum* Malaria J 2011 10 104 10.1186/1475-2875-10-104 PMC3112448 21527045

[B24] Londono-Renteria BL Eisele TP Keating J James MA Wesson DM Antibody response against *Anopheles albimanus* (Diptera: Culicidae) salivary protein as a measure of mosquito bite exposure in Haiti J Med Entomol 2010 47 1156 1163 10.1603/ME09240 21175067

[B25] Remoue F Cisse B Ba F Sokhna C Herve JP Boulanger D Simondon F Evaluation of the antibody response to *Anopheles* salivary antigens as a potential marker of risk of malaria Trans R Soc Trop Med Hyg 2006 100 363 370 10.1016/j.trstmh.2005.06.032 16310235

[B26] Balenghien T Fouque F Sabatier P Bicout DJ Horse-, bird-, and human-seeking behavior and seasonal abundance of mosquitoes in a West Nile virus focus of southern France J Med Entomol 2006 43 936 946 10.1603/0022-2585(2006)43[936:HBAHBA]2.0.CO;2 17017231

[B27] Toprak S Ozer N Distribution of sand fly (Diptera: Psychodidae) species and efficiency of capturing methods in Sanliurfa province, Turkey J Med Entomol 2007 44 23 28 10.1603/0022-2585-44.1.23 17294917

[B28] Rizzo C Ronca R Fiorentino G Mangano VD Sirima SB Nebie I Petrarca V Modiano D Arca B Wide cross-reactivity between *Anopheles gambiae* and *Anopheles funestus* SG6 salivary proteins supports exploitation of gSG6 as a marker of human exposure to major malaria vectors in tropical Africa Malaria J 2011 10 206 10.1186/1475-2875-10-206 PMC3160432 21794142

[B29] Lombardo F Ronca R Rizzo C Mestres-Simon M Lanfrancotti A Curra C Fiorentino G Bourgouin C Ribeiro JM Petrarca V Ponzi M Coluzzi M Arca B The *Anopheles gambiae* salivary protein gSG6: an anopheline-specific protein with a blood-feeding role Insect Biochem Mol Biol 2009 39 457 466 10.1016/j.ibmb.2009.04.006 19442731 PMC3740408

[B30] Poinsignon A Cornelie S Mestres-Simon M Lanfrancotti A Rossignol M Boulanger D Cisse B Sokhna C Arca B Simondon F Remoue F Novel peptide marker corresponding to salivary protein gSG6 potentially identifies exposure to *Anopheles* bites PLoS One 2008 3 e2472 10.1371/journal.pone.0002472 18575604 PMC2427200

[B31] Rizzo C Ronca R Fiorentino G Verra F Mangano V Poinsignon A Sirima SB Nebie I Lombardo F Remoue F Coluzzi M Petrarca V Modiano D Arca B Humoral response to the *Anopheles gambiae* salivary protein gSG6: a serological indicator of exposure to Afrotropical malaria vectors PLoS One 2011 6 e17980 10.1371/journal.pone.0017980 21437289 PMC3060095

[B32] Poinsignon A Samb B Doucoure S Drame PM Sarr JB Sow C Cornelie S Maiga S Thiam C Rogerie F Guindo S Hermann E Simondon F Dia I Riveau G Konate L Remoue F First attempt to validate the gSG6-P1 salivary peptide as an immuno-epidemiological tool for evaluating human exposure to *Anopheles funestus* bites Trop Med Int Health 2010 15 1198 1203 10.1111/j.1365-3156.2010.02611.x 20723184

[B33] Poinsignon A Cornelie S Ba F Boulanger D Sow C Rossignol M Sokhna C Cisse B Simondon F Remoue F Human IgG response to a salivary peptide, gSG6-P1, as a new immuno-epidemiological tool for evaluating low-level exposure to *Anopheles* bites Malaria J 2009 8 198 10.1186/1475-2875-8-198 PMC2733152 19674487

[B34] Gaayeb L Sarr JB Ndiath MO Hanon JB Debrie AS Seck M Schacht AM Remoue F Hermann E Riveau G Seroprevalence of pertussis in senegal: a prospective study PLoS One 2012 7 e48684 10.1371/journal.pone.0048684 23119090 PMC3485356

[B35] Ndiath MO Sarr JB Gaayeb L Mazenot C Sougoufara S Konate L Remoue F Hermann E Trape JF Riveau G Sokhna C Low and seasonal malaria transmission in the middle Senegal River basin: identification and characteristics of *Anopheles* vectors Parasit Vectors 2012 5 21 10.1186/1756-3305-5-21 22269038 PMC3274455

[B36] Thwing JI Perry RT Townes DA Diouf MB Ndiaye S Thior M Success of Senegal’s first nationwide distribution of long-lasting insecticide-treated nets to children under five - contribution toward universal coverage Malaria J 2011 10 86 10.1186/1475-2875-10-86 PMC3083382 21489278

[B37] Gillies MT De Meillon D The anophelinae of africa south of the sahara (ethiopian zoogeographical region) 1968 Johannesburg: South African Institute for Medical Research

[B38] Drame PM Poinsignon A Besnard P Le Mire J Dos-Santos MA Sow CS Cornelie S Foumane V Toto JC Sembene M Boulanger D Simondon F Fortes F Carnevale P Remoue F Human antibody response to *Anopheles gambiae* saliva: an immuno-epidemiological biomarker to evaluate the efficacy of insecticide-treated nets in malaria vector control AmJTrop Med Hyg 2010 83 115 121 10.4269/ajtmh.2010.09-0684 PMC2912587 20595489

[B39] Ribeiro JM Seulu F Abose T Kidane G Teklehaimanot A Temporal and spatial distribution of anopheline mosquitos in an Ethiopian village: implications for malaria control strategies Bull World Health Organ 1996 74 299 305 8789928 PMC2486923

[B40] Schäfer ML Influence of landscape structure on mosquitoes (Diptera: Culicidae) and dystiscids (Coleoptera: Dystiscidae) at five spatial scales in Swedish wetlands Wetlands 2006 26 57 68 10.1672/0277-5212(2006)26[57:IOLSOM]2.0.CO;2

[B41] Staedke SG Nottingham EW Cox J Kamya MR Rosenthal PJ Dorsey G Short report: proximity to mosquito breeding sites as a risk factor for clinical malaria episodes in an urban cohort of Ugandan children AmJTrop Med Hyg 2003 69 244 246 14628938

[B42] Van Der Hoek W Konradsen F Amerasinghe PH Perera D Piyaratne MK Amerasinghe FP Towards a risk map of malaria for Sri Lanka: the importance of house location relative to vector breeding sites Int J Epidemiol 2003 32 280 285 10.1093/ije/dyg055 12714550

[B43] Drame PM Machault V Diallo A Cornelie S Poinsignon A Lalou R Sembene M Dos Santos S Rogier C Pages F Le Hesran JY Remoue F IgG responses to the gSG6-P1 salivary peptide for evaluating human exposure to *Anopheles* bites in urban areas of Dakar region Senegal. Malaria J 2012 11 72 10.1186/1475-2875-11-72 PMC3337805 22424570

[B44] Port GR Boreham PFL Bryan JH The relationship of host size to feeding by mosquitoes of the *Anopheles gambiae* Giles complex (Diptera: Culicidae) Bull Entomol Res 1980 70 133 144 10.1017/S0007485300009834

[B45] Smith T Killeen G Lengeler C Tanner M Relationships between the outcome of *Plasmodium falciparu*m infection and the intensity of transmission in Africa AmJTrop Med Hyg 2004 71 80 86 15331822

[B46] Fontaine A Pascual A Orlandi-Pradines E Diouf I Remoue F Pages F Fusai T Rogier C Almeras L Relationship between exposure to vector bites and antibody responses to mosquito salivary gland extracts PLoS One 2011 6 e29107 10.1371/journal.pone.0029107 22195000 PMC3237593

[B47] Drame PM Poinsignon A Besnard P Cornelie S Le Mire J Toto JC Foumane V Dos-Santos MA Sembene M Fortes F Simondon F Carnevale P Remoue F Human antibody responses to the *Anopheles* salivary gSG6-P1 peptide: a novel tool for evaluating the efficacy of ITNs in malaria vector control PLoS One 2010 5 e15596 10.1371/journal.pone.0015596 21179476 PMC3001874

[B48] Orlandi-Pradines E Almeras L Denis de Senneville L Barbe S Remoue F Villard C Cornelie S Penhoat K Pascual A Bourgouin C Fontenille D Bonnet J Corre-Catelin N Reiter P Pages F Laffite D Boulanger D Simondon F Pradines B Fusai T Rogier C Antibody response against saliva antigens of *Anopheles gambiae* and Aedes aegypti in travellers in tropical Africa Microbes Infect 2007 9 1454 1462 10.1016/j.micinf.2007.07.012 17913537

[B49] Billingsley PF Baird J Mitchell JA Drakeley C Immune interactions between mosquitoes and their hosts Parasite Immunol 2006 28 143 153 10.1111/j.1365-3024.2006.00805.x 16542316

[B50] Kalluri S Gilruth P Rogers D Szczur M Surveillance of arthropod vector-borne infectious diseases using remote sensing techniques: a review PLoS Pathog 2007 3 1361 1371 17967056 10.1371/journal.ppat.0030116 PMC2042005

[B51] Robert V Boudin C Biology of man-mosquito *Plasmodium* transmission Bull Soc Pathol Exot 2003 96 6 20 12784587

